# NKp30 Receptor Upregulation in Salivary Glands of Sjögren’s Syndrome Characterizes Ectopic Lymphoid Structures and Is Restricted by Rituximab Treatment

**DOI:** 10.3389/fimmu.2021.706737

**Published:** 2021-09-14

**Authors:** Elena Pontarini, Elisabetta Sciacca, Sofia Grigoriadou, Felice Rivellese, Davide Lucchesi, Liliane Fossati-Jimack, Rachel Coleby, Farzana Chowdhury, Francesca Calcaterra, Anwar Tappuni, Myles J. Lewis, Martina Fabris, Luca Quartuccio, Silvia Della Bella, Simon Bowman, Costantino Pitzalis, Domenico Mavilio, Salvatore De Vita, Michele Bombardieri

**Affiliations:** ^1^Centre for Experimental Medicine and Rheumatology, William Harvey Research Institute, London, United Kingdom; ^2^Institute of Dentistry, Barts and the London School of Medicine and Dentistry, London, United Kingdom; ^3^Laboratory of Clinical and Experimental Immunology, Istituti di Ricovero e Cura a Carattere Scientifico (IRCCS) Humanitas Research Hospital, Rozzano, Italy; ^4^Department of Medical Biotechnologies and Translational Medicine, University of Milan, Milan, Italy; ^5^Istituto Di Patologia Clinica, Azienda Sanitaria Universitaria Integrata di Udine (ASUID), Udine, Italy; ^6^Clinic of Rheumatology, Department of Medicine (DAME), University of Udine, School of Rheumatology, Academic Hospital “Santa Maria della Misericordia”, Udine, Italy; ^7^National Institute for Health Research (NIHR) Birmingham Biomedical Research Centre, University Hospitals Birmingham National Health System (NHS) Foundation Trust, Birmingham, United Kingdom

**Keywords:** Sjögren’s syndrome, salivary gland, NK cell, NKp30, epithelial cell, B7/H6

## Abstract

Primary Sjögren’s syndrome (pSS) is a chronic autoimmune disease resulting from the inflammatory infiltration of exocrine glands, mainly salivary and lacrimal glands, leading to secretory dysfunction and serious complications including debilitating fatigue, systemic autoimmunity, and lymphoma. Like other autoimmune disorders, a strong interferon (IFN) signature is present among subsets of pSS patients, suggesting the involvement of innate immunity in pSS pathogenesis. *NCR3*/NKp30 is a natural killer (NK) cell-specific activating receptor regulating the cross talk between NK and dendritic cells including type II IFN secretion upon NK-cell activation. A genetic association between single-nucleotide polymorphisms (SNPs) in the *NCR3*/NKp30 promoter gene and a higher susceptibility for pSS has been previously described, with pSS patients most frequently carrying the major allele variant associated with a higher NKp30 transcript and IFN-γ release as a consequence of the receptor engagement. In the present study, we combined RNA-sequencing and histology from pSS salivary gland biopsies to better characterize NKp30 (*NCR3*) and its ligand B7/H6 (*NCR3LG1*) in pSS salivary gland tissues. Levels of *NCR3*/NKp30 were significantly increased both in salivary glands and in circulating NK cells of pSS patients compared with sicca controls, especially in salivary glands with organized ectopic lymphoid structures. In line with this observation, a strong correlation between *NCR3*/NKp30 levels and salivary gland infiltrating immune cells (CD3, CD20) was found. Furthermore, *NCR3*/NKp30 levels also correlated with higher IFN-γ, Perforin, and Granzyme-B expression in pSS SGs with organized ectopic lymphoid structures, suggesting an activation state of NK cells infiltrating SG tissue. Of note, NKp30+ NK cells accumulated at the border of the inflammatory *foci*, while the NKp30 ligand, B7/H6, is shown to be expressed mainly by ductal epithelial cells in pSS salivary glands. Finally, immunomodulatory treatment, such as the B-cell depleting agent rituximab, known to reduce the infiltration of immune cells in pSS SGs, prevented the upregulation of *NCR3*/NKp30 within the glands.

## Introduction

Primary Sjögren’s syndrome (pSS) is a chronic autoimmune exocrinopathy characterized by an immune response within the salivary and lachrymal glands leading to the loss of secretory function of exocrine glands, or sicca syndrome (1). Sjögren’s syndrome (pSS) is commonly associated with the development of circulating autoantibodies, such as those targeting the ribonucleoproteins Ro/SSA and La/SSB (i.e., anti-Ro/SSa and anti-La/SSB autoantibodies), and rheumatoid factor (RF) (2). Besides oral and ocular manifestations, and the development of circulating autoantibodies, the main histopathologic hallmark of the disease is the development of lymphomonocytic infiltration within the glands, with inflammatory aggregates (*foci*) organized around central salivary and lachrymal ducts (3). Although the ductal epithelial cells (ECs) are the target of inflammation within the salivary glands (SGs), they also act as unconventional antigen-presenting cells, expressing immuno-modulatory molecules able to promote immune-cell recruitment and activation, namely, dendritic cells (DCs), natural killer (NK) cells, and T cells, which results in EC apoptosis (4).

he role of adaptive immunity in the pathogenesis of pSS has been well established; however, far less is known about the contribution of innate immunity and its interaction with adaptive immunity. Earlier gene expression profiling studies showed upregulation of a type I interferon (IFN) signature in patients with pSS (5–9), suggesting the involvement of the innate arm of the immune system in the disease pathogenesis. This occurs mainly in response to the enhanced apoptosis of SG EC, which is thought to be triggered following a viral/infectious insult (10). Animal models of pSS also indicate a crucial role for type II IFN in the disease pathogenesis (11, 12).

The role of NK cells in pSS remains unclear. Although animal models of pSS have not directly implicated NK cells in disease pathogenesis, resident NK cells as well as NK cells infiltrating from the peripheral compartment are readily activated in experimental sialoadenitis (13, 14). While most of the data published to date that investigated the number and/or the functional impairment of the NK cell compartment in patients with pSS led to contradictory results (15–17), recent publications suggest a critical role of NK cells as mediators of both type II and type I IFN functions (18, 19).

NK cell activation is regulated by a delicate balance between activating and inhibitory receptors and triggered by the engagement of their activating receptors with their cognate ligands. In the context of pSS, NK cell activation is thought to be facilitated by engagement of the natural cytotoxicity receptor (NCR) NKp30 with its ligand B7 homolog 6 (B7/H6), also known as natural killer cell cytotoxicity receptor 3 ligand 1 (*NCR3LG1*) (18). B7/H6 is a human-specific B7 family member that binds to and activates the NKp30 receptor. B7/H6 is typically not expressed on normal human tissues, but it has been described in primary tumors or upregulated under inflammatory conditions, mainly induced upon stimulation by ligands of toll-like receptors or pro-inflammatory cytokines (20). Several B7 superfamily costimulatory molecules, which also include CD80 (B7.1), CD86 (B7.2) or ICOSL, and PDL1 (21), are enhanced on the surface of SG EC in pSS patients, supporting their function as antigen-presenting cells, which results in priming of DC and T-cell activation (22–25).

It has been postulated that the inflammatory environment generated within the SGs following the initial insult, presumed viral or environmental, results in the upregulation of the NK cell ligand B7/H6 on SG EC (26) and DC, leading to the activation of NK cells, which in turn produce type II IFN (mainly IFN-γ). DC, in particular plasmacytoid DC, can also produce type I IFN and interleukin-12 (IL-12), a potent NK and T-cell activator and IFN-γ inducer, which perpetuates the local inflammation (that leads to EC destruction and exposure of autoantigens) (19).

More recently, B7/H3 was also found to be upregulated on SG EC in pSS patients, promoting inflammation by activating the NF-κB pathway which results in increased levels of interleukin-6 (IL-6) and tumor necrosis factor-alpha (TNFα) enhancing the apoptosis of SG EC (27). TNFα has been shown to be the main inflammatory stimuli able to induce the B7/H6 upregulation in the SG EC cell line (18).

A case–control study found an association between pSS and two single-nucleotide polymorphisms (SNPs) (rs11575837 and rs27366191) in the promoter region of the NCR3 locus, encoding for the NKp30 activating receptor (18). An independent study in a Scandinavian cohort confirmed the association between the rs11575837 SNP and anti-Ro/SSA and anti-La/SSB positivity in patients with pSS. The rs11575837 SNP was shown to be protective for pSS development, as it was reported to be less frequent in patients with pSS compared to controls, and it was linked to a reduced transcription of NCR3. pSS patients were shown to carry most frequently the major allele for rs11575837 SNP, associated with a higher NKp30 transcript and IFN-γ release, as a consequence of the receptor engagement. In line with genetics findings, NKp30 was described to be upregulated on circulating NK cells in pSS patients (18).

Despite the association between the SNPs in the NCR3/NKp30 promoter gene and pSS susceptibility, alongside the upregulation of NKp30 on circulating NK cells, the expression of the receptor and its ligand within pSS SG is still poorly characterized.

To this aim, in the present study, we combined RNA-sequencing and SG histology as well as peripheral blood flow cytometry to characterize the expression of NKp30 and its ligand B7/H6 in pSS patients. Furthermore, we studied the effects of an immunomodulatory treatment with the B-cell depleting agent, rituximab, on their expression at tissue level.

## Materials and Methods

### Patient Samples

Samples were collected from healthy donors (HDs), patients with pSS, and non-specific chronic sialadenitis (sicca, NSCS). The diagnosis of pSS was made according to the 2002 revised classification criteria of the American–European Consensus Group (28). Demographic, clinical, and laboratory data of the patients enrolled in this study are provided in **Supplementary Table S1**.

For flow-cytometry analysis, blood was collected from patients with pSS (*n* = 23) and from healthy donors (HDs) (*n* = 20), respectively, from the Rheumatology Clinic, University of Udine (Italy) and Humanitas Clinical and Research Center, Milan (Italy). pSS patients were selected based on their salivary histopathology, recruiting 11 patients with labial salivary gland inflammatory infiltration, 7 with myoepithelial sialadenitis (MESA), and 5 non-Hodgkin’s MALT lymphoma in the parotid salivary glands. The HDs consisted of women without symptoms or signs of xerostomia or xerophthalmia, or any history of autoimmune rheumatic diseases.

For RNA sequencing analysis, labial SG biopsies from patients with pSS (*n* = 24) and NSCS (*n* = 17) were obtained from the Dental Clinic, Barts and The London School of Medicine and Dentistry at Barts Health NHS Trust.

From the TRACTISS trial (ISRCTN: 65360827/European Clinical Trials database no. 2010-021430-64) cohort (29, 30), 26 pSS patients gave consent for labial SG biopsies at baseline, weeks 16 and 48. Following randomization to Rituximab or placebo, patients received two 1,000-mg cycles of Rituximab/placebo at baseline and week 24.

All patients gave written informed consent and approval was obtained by local ethics committees.

### RNA Extraction and Bulk RNA Sequencing on Labial SG Tissue

RNA was extracted using RNeasy Micro Kit (Qiagen) following the manufacturer’s instructions. The RNA samples were quantified using Qubit 2.0 Fluorometer (Invitrogen) and RNA integrity was checked with Agilent TapeStation (Agilent Technologies).

RNA sequencing libraries were prepared using the NEBNext Ultra RNA Library Prep Kit for Illumina. Briefly, mRNA was first enriched with Oligod(T) beads. Enriched mRNAs were fragmented for 15 min at 94°C. First-strand and second-strand cDNA were subsequently synthesized. cDNA fragments were end repaired and adenylated at 3’ends, and universal adapters were ligated to cDNA fragments, followed by index addition and library enrichment by PCR with limited cycles. The sequencing library was validated on the Agilent TapeStation (Agilent Technologies) and quantified by using Qubit 2.0 Fluorometer (Invitrogen) as well as by quantitative PCR (KAPA Biosystems).

Libraries were sequenced on Illumina HiSeq 4000, using 2×150 bp paired end configuration, 50 million reads/sample. One mismatch was allowed for index sequence identification (Genewiz).

### Immunohistochemistry on SG Tissue

Immunohistochemistry (IHC) was performed on formalin-fixed and paraffin-embedded tissue sections of labial SG biopsies. After deparaffinization, sections were pretreated for 15 min in the pressure cooker with tris-EDTA buffer (10 mM tris, 1 mM EDTA, pH 9, Dako) for NKp30 and B7/H6, citrate buffer (pH 6.0, Dako) for CD20, CD138, and CD3, or Proteinase-K (Dako) for CD21 antigens. Endogenous peroxidase activity was inhibited with 3% hydrogen peroxide (Dako) for 10 min. Tissue sections were incubated with the primary antibodies (**Supplementary Table S2**) for 1 h, followed by HRP-conjugated secondary antibody and developed with 3,3′-Diaminobenzidine (DAB, Dako). The sections were counterstained with Harris’s hematoxylin and mounted. Positive controls for both NKp30 and B7/H6 are shown in **Supplementary Figure S1**.

The assessment of the inflammation in the labial salivary gland biopsies and the histological identification of ELS is based on the IHC staining for T cells (CD3), B cells (CD20), the presence of follicular dendritic cell (FDC) network (CD21), and plasma cells (CD138). The ELS is defined as at least one infiltrate with clear B/T cell compartmentalization in discrete areas and presence of FDC within the B-cell area, suggestive of germinal center presence (**Supplementary Figure S2**). Inflammation in the labial salivary gland is scored with a semi-quantitative grading system, which classifies the periductal inflammatory aggregates into four histologic groups (from 0 to 3) according to the size and the degree of lymphoid organization based on B- and T-cell segregation and presence of CD21+ FDC network (31).

### Blood Sample Processing

Venous blood samples were collected in potassium-ethylenediaminetetraacetic acid (K-EDTA) anticoagulant. The samples were processed within 24 h of collection. Peripheral blood mononuclear cells (PBMCs) were isolated after Ficoll density gradient centrifugation. The PBMCs were washed twice with PBS and then stored at −80°C in freezing medium [90% fetal bovine serum (FBS), 10% dimethylsulfoxide (DMSO)] until further analysis.

### Flow Cytometry

Flow-cytometry analysis was performed on 43 frozen samples of PBMCs. After thawing, cells were stained with Zombie Aqua Live/Dead kit (BioLegend) for 15 min, washed, and incubated for 10 min with human Fc TruStain FcX (BioLegend). Cells were stained for surface antigens combined in a seven-color panel (CD14, CD3, CD20, CD56, CD16, and NK-receptor). Cells were split in up to five tubes, one for each NK-receptor (NKp46, NKp44, NKp30, NKG2D, and DNAM-1) with 1 million cells per tube. Antibodies used are listed in **Supplementary Table S3**. Cells were acquired using a FACS Canto II (BD Biosciences) flow cytometer and analyzed with FlowJo V.2 software.

### Statistical and Bioinformatics Analysis

Differences in quantitative variables between two groups were analyzed by Mann–Whitney two-tailed *U* test or Student’s *t*-test, as appropriate after assessing the distribution of the data using the Shapiro–Wilk test and QQ plots. For multiple comparison, Kruskal–Wallis test with Dunn’s post-hoc correction or one-way ANOVA was used. *p*-values less than 0.05 were considered significant. Statistical analyses, including the analysis of flow-cytometry data, were performed using GraphPad Prism 9.0 (GraphPad Software, La Jolla, CA, USA).

**Figure 1:**
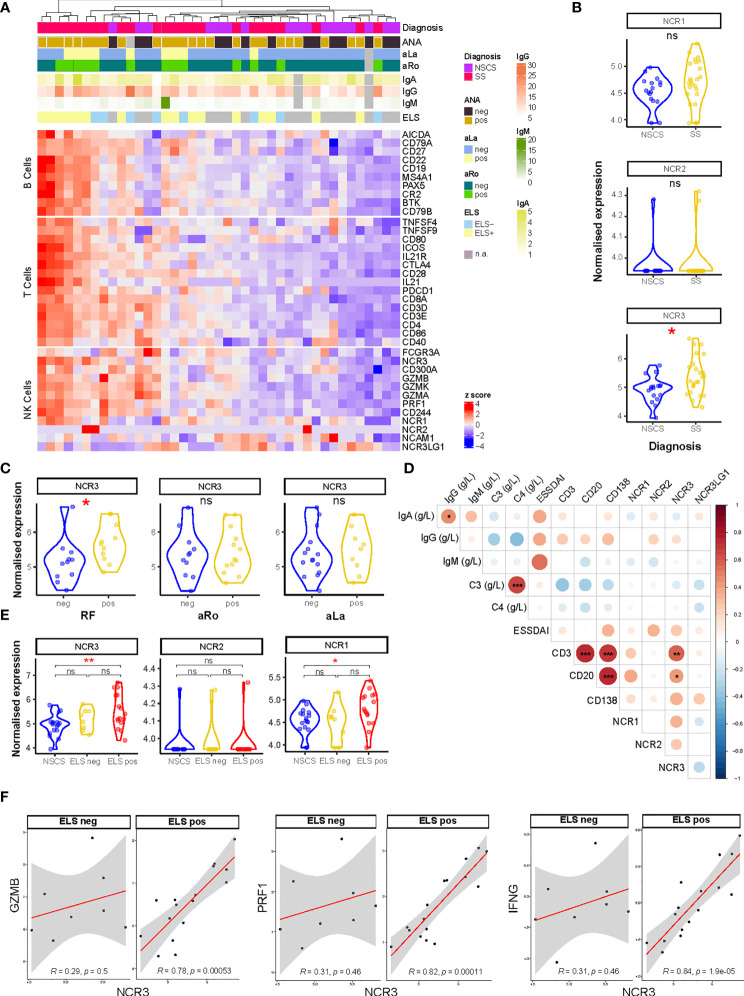
NCR3 gene is upregulated in pSS salivary glands with higher inflammatory infiltration. **(A)** Heatmap showing Z-score of NK, B-, and T-cell related genes from RNA sequencing analysis of labial salivary gland biopsies of Sicca (*n* = 17) and pSS (*n* = 24) patients. Color sets on top of the heatmap identify the presence (pos) or absence (neg) of circulating anti-nuclear (ANA), anti-Ro (aRo), and anti-La (aLa) antibodies, ectopic lymphoid structures (ELS) in the glands, and circulating immunoglobulin (Ig) levels (IgG, IgA, and IgM). Each square is a sample/patient. **(B)** Expression on natural cytotoxicity receptor (NCR) genes: *NCR1* (NKp46), *NCR2* (NKp44), and *NCR3* (NKp30) between NSCS (sicca) and pSS salivary glands. Mann–Whitney *U t*-test statistics. **(C)** *NCR3* (NKp30) gene expression level in SS patients segregated by the presence (pos) or absence (neg) of circulating auto-antibodies, such as rheumatoid factor (RF), anti-SSA/Ro (aRo), and anti-SSB/La (aLa). Mann–Whitney *U t*-test statistics. **(D)** Correlation matrix between *NCR1* (NKp46), *NCR2* (NKp44), *NCR3* (NKp30), *NCR3LG1* (B7/H6) gene expression level, circulating level of immunoglobulins (IgG, IgA, IgM), complement (C3, C4), EULAR Sjögren’s syndrome (SS) disease activity index (ESSDAI), and histological semi-quantitative score for CD3, CD20, and CD138. Graph shows NSCS and pSS patients. Spearman correlation coefficient, *R* (color denotes the strength of the correlation) and *p*-value, FDR correction for multiple comparison. *p < 0.05, **p < 0.01, ***p < 0.001. **(E)** *NCR3* (NKp30) gene expression level in NSCS (sicca) and pSS salivary glands, segregated by the presence (ELS pos) or absence (ELS neg) of ectopic lymphoid structures within SG. Statistical analysis by Kruskal–Wallis test with Dunn’s multiple comparison correction. **(F)** Spearman correlation analysis between *NCR3* with *GZMB, PRF1*, and *IFNG* in pSS patients segregated for the presence of ELS. Spearman correlation coefficient, *R* and *p*-value, *p < 0.05, **p < 0.01, ***p < 0.001. NSCS, non-specific chronic sialadenitis; SS, Sjogren’s syndrome; ELS, ectopic lymphoid structures; ns, not significant.

RNA sequencing data were analyzed with R (v.4.0.4) software. The heatmap with unsupervised clustering was generated using the ComplexHeatmap (v.2.6.2) package using Euclidean distance and complete linkage method for clustering, annotating pre-selected genes of interest (**Figure 1**). Violin plots were generated through the ggplot2 package.

The differential gene expression analysis was evaluated using the R package DESeq2 (v.1.30.1). False discovery rate (FDR) was applied using Storey’s *q*-value with a cutoff of *q* < 0.05 used to significantly define differentially expressed genes (DEGs). In this analysis, pseudogenes were removed, and the linear model was adjusted by gender used as covariate (**Figures 1** and **4**). The DEGs for **Figure 1A** analysis are listed in **Supplementary Table S4**.

For the TRACTISS cohort data (**Figure 4**), a gene level longitudinal analysis was performed fitting a mixed effects linear model through the R package glmmSeq (v.0.1.0). Gene dispersions were calculated through the following formula:


$$Dispersion_{i}=(variance_{i}-mean_{i})/mean_{i}^{2}$$

Size factors were estimated using the estimateSizeFactorsForMatrix function from DESeq2 (v.1.30.1).

RNA-seq data have been deposited in the ArrayExpress database at EMBL-EBI (www.ebi.ac.uk/arrayexpress) under accession number E-MTAB-10517.

## Results

### NKp30 Is Upregulated in Sjogren’s Syndrome Salivary Glands

Bulk-RNA sequencing analysis was performed on labial salivary glands biopsies from NSCS and pSS patients. Unsupervised clustering showed a segregation of NK cell genes together with B- and T-cell gene signatures in pSS patients (**Figure 1A**), suggesting an enrichment of NK cells in salivary glands with a higher inflammatory infiltration. The list of DEGs is reported in **Supplementary Table S4**. Focusing on the expression of the natural cytotoxicity receptor (NCR) family, we looked at the expression level of *NCR1* (NKp46, CD335), *NCR2* (NKp44, CD336), and *NCR3* (NKp30, CD337). Although these molecules have no homology, they have been grouped as NCRs based on the similarities in their expression profile, their oligomeric structures, and their function (32).

*NCR3* gene showed a significantly higher expression in pSS salivary gland tissues compared to the NSCS glands, while *NCR1* and *NCR2* genes did not show a differential expression between the two groups (**Figure 1B**). NCR3 upregulation in pSS patients compared to NSCS within salivary gland tissue was confirmed using qPCR (**Supplementary Figure S3B**). *NCR3* expression was higher in pSS patients with circulating autoantibodies, such as rheumatoid factor (RF), but not anti-SSA/Ro and anti-SSB/La (**Figure 1C**). No significant correlation was found between *NCR3* and clinical parameters reflecting B-cell hyper-activation, such as peripheral blood immunoglobulins (IgG, IgM, and IgA) or complement (C3 and C4). Of note, however, *NCR3* showed strong correlations with salivary gland inflammatory markers, such as semi-quantitative scores for B (CD20) and T (CD3) cells in pSS labial salivary glands (**Figure 1D**). These correlations were specific for NCR3, as they were not observed for *NCR1* and *NCR2* (**Figure 1D**).

The quantification of salivary gland infiltration of B (CD20) and T (CD3) cells also identified inflammatory infiltrate organization in ectopic lymphoid structures (ELS), (**Supplementary Figure S2**). Based on the salivary gland histology, pSS patients were classified as ELS positive or negative, according to the presence or absence of segregated *foci* in the salivary glands (33). An upregulation of *NCR3* (alongside *NCR1*) characterized salivary glands with a higher inflammatory infiltration and ELS organization (**Figure 1E**), confirmed by qPCR (**Supplementary Figure S3C**). *NCR3* showed a higher expression among the two NCR receptors (**Figure 1E**). Next, we analyzed gene–gene correlations and observed a strong, positive correlation between *NCR3* and *GZMB, PRF1*, and *IFNG*, encoding respectively for Granzyme-B, Perforin, and IFN-γ. Importantly, the correlations were exclusively present in pSS salivary glands with ELS (**Figure 1F**). On the other hand, *NCR2* did not correlate with these NK cell effector mediators and *NCR1* showed a weak correlation with *PRF1* and *IFNG* (**Supplementary Figure S4A**), suggesting that NK cell activation in pSS SGs is mainly driven by NKp30 engagement.

### NKp30 and B7/H6 Localization in Salivary Glands

**Figure 2:**
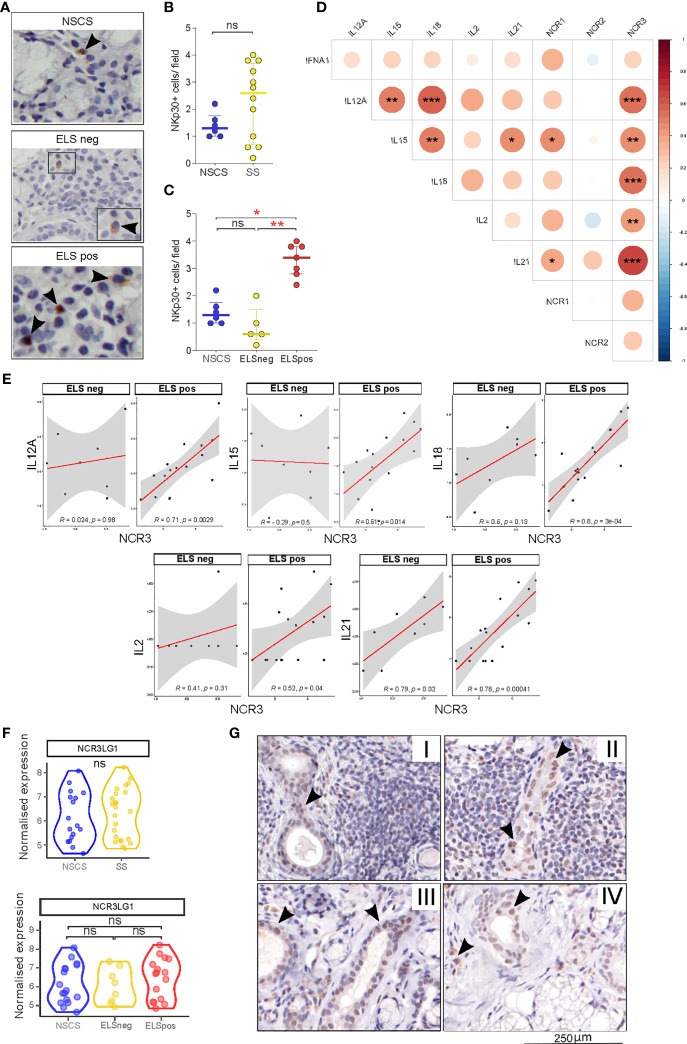
NKp30 and B7/H6 localization in salivary gland tissues. **(A)** Representative images of immunohistochemistry staining for NKp30 in NSCS (*n* = 6) and SS (*n* = 12) labial salivary gland biopsies. Arrowhead: NKp30+ cells. **(B, C)** Quantification (mean count per field) of NKp30+ cells in labial SG biopsies of NSCS and SS **(B)**, segregated by the presence of ELS **(C)**. Mann–Whitney *U t*-test statistics in **(B)** and Kruskal–Wallis test with Dunn’s multiple comparison correction **(C)**. All graphs represent median with interquartile range. *p < 0.05, **p < 0.01, ***p < 0.001. **(D)** Correlation matrix between *NCR1* (NKp46), *NCR2* (NKp44), and *NCR3* (NKp30) with *IL12A, IL15, IL18, IL2*, and *IL21* gene expression levels from bulk-RNA sequencing of SG tissues. Graph shows NSCS (*n* = 17) and pSS (*n* = 24) patients. Spearman correlation coefficient, *R* (color denotes the strength of the correlation) and *p*-value, FDR correction for multiple comparison. *p < 0.05, **p < 0.01, ***p < 0.001. **(E)** Spearman correlation analysis between NCR3 with IL12A, IL15, IL18, IL2 and IL21 in pSS patients (n = 24) segregated by the presence of ELS. Spearman correlation coefficient, *R* and *p*-value, *p < 0.05, **p < 0.01, ***p < 0.001. **(F)** *NCR3LG1* (B7/H6) gene expression level in NSCS (*n* = 17) and pSS (*n* = 24) salivary glands (top graph), and NSCS with pSS segregated by the presence (ELS pos) or absence (ELS neg) of ectopic lymphoid structures within SG (bottom graph). Mann–Whitney *U t*-test statistics and Kruskal–Wallis test with Dunn’s multiple comparison correction respectively. **(G)** Representative images of immunohistochemistry staining for B7/H6 in labial salivary gland biopsies of SS with different degrees of inflammation (I, II, and III) and NSCS (IV). Arrowhead: B7/H6 + cells. ns, not significant.

Having observed an upregulation of *NCR3* gene expression in pSS salivary glands, we then looked at the localization of NKp30+ NK cells and its ligand B7/H6 in SG tissues, stratified according to the severity of the inflammatory infiltrates. Staining for NKp30 (**Figure 2A**) revealed increased numbers of tissue-NK cells in pSS salivary glands displaying a higher degree of inflammation and ELS formation (**Figures 2B, C**), compared to NSCS patients. In pSS patients, these NKp30+ NK cells accumulated mainly at the border of the inflammatory *foci* (**Figure 2A**).

Cytokines including IL-12, IL-15, and IL-18 are critical regulators of NK cell activation, while others such as IL-2 and IL-21 have been described to modulate the receptor repertoire of NK cells, including NCR members such as NKp46 (34). We wondered whether similar pro-inflammatory stimuli might be responsible for NKp30 (NCR3) and to a lesser extent NKp46 (NCR1) upregulation within pSS SG. Accordingly, we observed a strong correlation of *NCR1* with IL-15 and IL-21 in pSS SGs (**Figure 2D**), confirming previous findings on the effect of IL-21 in inducing NKp46 upregulation on PBMCs (34). Interestingly, NCR3 strongly correlates with IL12-A, IL-15, IL-18, IL-2, and IL-21 in SGs tissue (**Figure 2D**). Stratifying the pSS cohort based on ELS organization, these correlations were only observed in SG with ELS (**Figure 1E**), with IL-18 and IL-21, the latter known to be associated with ELS organization (33), showing the strongest correlations. These correlations were not observed for NCR2 (**Supplementary Figure S4B**).

Looking at the expression of NKp30 receptor ligand (*NCR3LG1*, B7/H6), RNA sequencing did not show any differential expression between NSCS or pSS salivary gland tissues regardless of inflammatory infiltration (**Figure 2F**), suggesting a ubiquitous expression within salivary gland tissue. Consistent with the transcriptomic data, B7/H6 appeared to be expressed primarily by ductal epithelial cells (**Figure 2G**) in both NSCS and pSS salivary glands with and without inflammatory *foci*. Of note, B7/H6 expression was also found in some mononuclear cells, some of them morphologically identified as plasma cells, previously described only in cervical cancer (35) (**Supplementary Figure S1A**).

### NKp30 Upregulation on Circulating NK Cells Is Independent From Salivary Gland Histology

**Figure 3:**
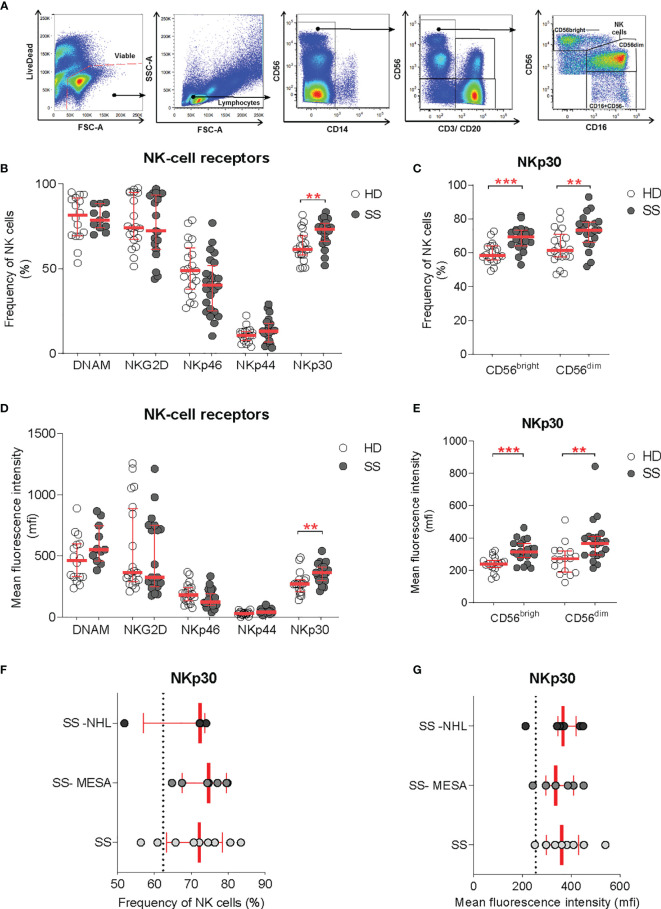
NKp30 expression on circulating NK cells. **(A)** Flow-cytometry gating strategy for the identification of NK cells and NK cell subsets (CD56dim and CD56bright) in the peripheral blood for receptor repertoire expression analysis. **(B–E)** Comparison of NK cell receptor expression between healthy donors (HDs) (*n* = 20) and SS (*n* = 23) PBMCs, expressed as frequency **(B, C)** and mean fluorescence intensity **(D, E)**, on total NK cells and NK cell subsets, respectively. **(F, G)** NKp30 expression on total NK cells [as frequency **(F)** and MFI **(G)**] in the pSS cohort segregated by SG histopathology: SG inflammatory infiltration with no feature of lymphomatous lesions (SS), myoepithelial sialadenitis (SS-MESA), and parotid non-Hodgkin’s MALT lymphoma (SS-NHL). The dotted line shows the average of the HD group. Each dot represents one sample/patient. Median with interquartile range in red. Mann–Whitney *U t*-test. **p < 0.01, ***p < 0.001.

Immuno-phenotypic characterization of circulating NK cells was performed on pSS patients and gender-matched controls. The NK-cell receptor repertoire was analyzed on total circulating NK-cell and NK subsets by flow cytometry. NK cells were identified as viable lymphocytes prior to the exclusion of CD14+ (monocytes), CD20+ (B cells), and CD3+ (T and NK-T cells) and gated on CD56+ population (NK cells). Based on the CD56 and CD16 expression, the main circulating NK cell subsets were identified as CD56bright (CD56++CD16-) and CD56dim (CD56+CD16+), respectively (**Figure 3A**).

Among all activating receptors analyzed, NKp30 was the only one upregulated on circulating NK cells in pSS patients compared with controls, whereas NKp46, NKp44, NKG2D, and DNAM-1 activating NK cell receptors were not significantly different. NKp30 upregulation was confirmed in terms of both frequency and receptor density (mean fluorescence intensity, MFI) (**Figures 3B, D**). NKp30 expression level was higher in the NK cell compartment overall, with an increased expression on both CD56bright and CD56dim subsets (**Figures 3C, E**).

Given the selective NKp30 upregulation in SG tissue of pSS and the NCR evolvement in tumor surveillance, we investigated whether the NKp30 expression on circulating NK cells (expressed both as frequency and MFI) was different according to the presence of SG inflammatory infiltration (regardless of the ELS organization), myoepithelial sialadenitis (MESA) pre-lymphomatous lesions, or non-Hodgkin’s MALT lymphoma in SS parotid SG. The NKp30 expression on peripheral NK cells was comparable between patients with salivary gland inflammatory infiltration with no feature of lymphomatous lesions, myoepithelial sialadenitis (MESA), or non-Hodgkin’s MALT lymphoma in the parotid SG (**Figures 3F, G**).

### Rituximab Prevents NKp30 Upregulation in Sjogren’s Syndrome Salivary Glands

As we showed that the NKp30 upregulation in the SG tissue is associated with higher SG infiltration with features of ectopic germinal centers (GC), we evaluated whether treatment with rituximab, which was shown to modulate GC response in the SG (36), could also affect NKp30 receptor and/or B7/H6 ligand expression within pSS salivary glands.

**Figure 4:**
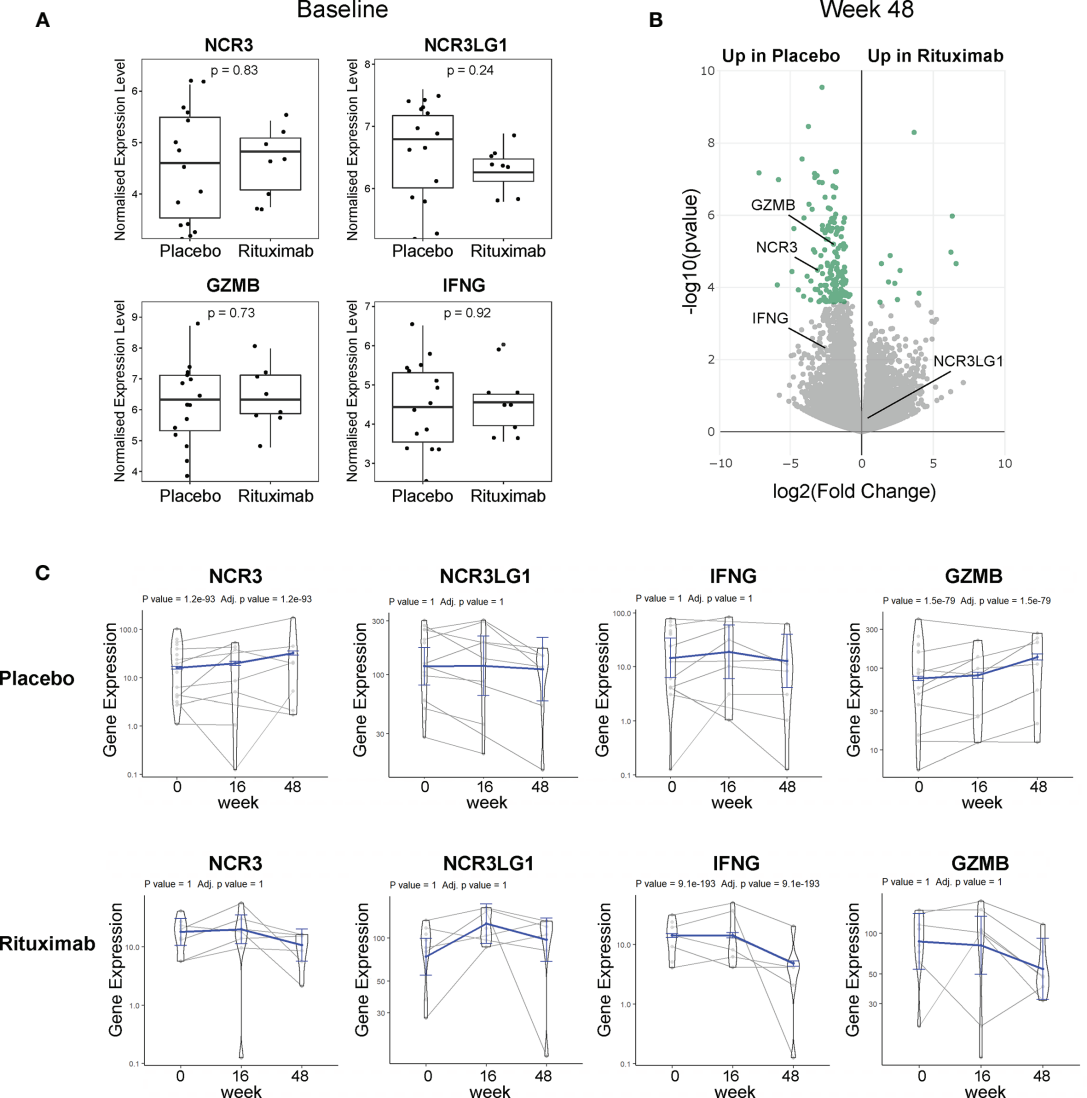
NKp30 and B7/H6 expression post-Rituximab treatment. **(A)** Baseline expression of *NCR3* (NKp30), *NCR3LG1* (B7/H6), *GZMB*, and *IFNG* between placebo and rituximab salivary glands. Mann–Whitney *U t*-test statistics. **(B)** Volcano plot of DEGs using DESeq2 comparing rituximab *versus* placebo patients after 48 weeks of treatment. Comparison between groups using Wald test and correcting for multiple testing Storey’s *q*-value (*q* < 0.05 = significant, shown in green). Positive values represent upregulation in rituximab and negative values denote downregulation in rituximab-treated patients compared to placebo. **(C)** Longitudinal analysis fitting a mixed effects linear model of *NCR3, NCR3LG1, IFNG, and GZMB* genes for rituximab (21 samples, 10 individuals) and placebo (29 samples, 15 individuals). The scatter plot shows the assessed normalized expression level of each sample over time overlaid by the fitted model (in blue) with 95% confidence intervals (fixed effects).

Available RNA sequencing data of labial SG biopsies from the TRACTISS cohort of pSS (29) were used for a longitudinal analysis of *NCR3* (NKp30) and *NCR3LG*1 (B7/H6) genes. When analyzing labial SG biopsies, no differences were observed in the expression of *NCR3*/NKp30, *NCR3LG1*/B7H6, *GZMB*, and *IFNG* at baseline between placebo and rituximab-treated patients (**Figure 4A**). Of note, in matched labial SG biopsies analysis at three different time points (baseline, week 16, and week 48), *NCR3* appears as one of the DEGs between placebo and rituximab at 48 weeks (**Figure 4B**). When looking at changes in the expression level in the three sequential biopsies (baseline, week 16, and week 48), *NCR3* expression increased over time in the placebo group, while a trend towards reduction was observed in the rituximab-treated group, with a similar pattern to *GZMB* and *IFNG* gene expression (**Figure 4C**). These data suggest that rituximab prevents the upregulation of NKp30 in pSS SG, while also reducing effector mediators of NK cell activation. On the contrary, NCR3LG1 (B7/H6) showed no changes over time within pSS SG tissue or as an effect of rituximab treatment. Rituximab prevents the worsening of SG inflammation. Placebo-treated labial SGs showed a worsening of inflammation highlighted by the increment of B-cell density, development of new FDC networks, and a higher ectopic GC prevalence at week 48, compared to RTX-treated patients (37).

## Discussion

Our findings focused on the NK cell-specific activating receptor NKp30 and its ligand B7/H6 within SGs, the target tissue of the autoimmune response in pSS. We showed a higher expression of the *NCR3* gene, which encodes for NKp30, in pSS SG biopsies compared with NSCS, which strongly correlates with the degree of glandular inflammation, as higher levels were specifically observed in patients with SG ELS. Moreover, using NKp30 staining, we were able to localize NKp30+ NK cells outside the inflammatory infiltrates within the glands.

Although we did not find a correlation between NKp30 expression and specific autoantibody production (SSA/Ro and anti-SSB/La) or peripheral blood immunoglobulins, both markers of B-cell hyperactivation, NKp30 was associated with the presence of ELS within SG. In addition, we demonstrated an activation status of the NK cells within the glands as evidenced by the upregulation of the genes encoding for the effector mediators perforin, granzyme-B, and IFN-γ. The production of perforin and granzyme-B is associated with a cytotoxic function of NK cells whereas IFN-γ defines a regulatory/inflammatory function (38).

Next, detailed immuno-phenotyping of circulating NK cells showed the selective upregulation only of the NKp30 activating receptor on both CD56bright and CD56dim NK cells, confirming a previous finding reporting an upregulation of NKp30 expression on circulating NK cells in pSS (26); this was independent of the severity of the local inflammation within SG.

Altogether, our results indicate that the natural cytotoxicity receptor (NCR) NKp30 is expressed in NK cells both in the peripheral blood and infiltrating SG tissues in SS patients.

The study by Rusakiewicz et al. (26) was the first to suggest the role of the NKp30 receptor in pSS (26). In this study, genetic polymorphisms (rs11575837, rs2736191) residing within the promoter region of NKp30 were associated with reduced gene transcription and function and reduced risk of the disease. In particular, the association between rs11575837 A allele and disease protection was even stronger among patients whose disease is characterized by specific autoantibody production (anti-Ro/SSA and anti-La/SSB). Conversely, the presence of the major G allele, which was more frequent in pSS patients than in controls, could lead to increased levels of NKp30 mRNA expression favoring IFN-γ secretion upon triggering by ligands.

The inflammatory stimuli inducing NKp30 upregulation in SS remains to be unraveled. *In vitro* studies on human NK cells showed that the upregulation of NK cell receptor part of the NCR family, such as NKp46, and the production of effector molecules (perforin and granzyme-A and B) is IL-21 and, to a lesser extent, IL-2 dependent, (34) suggesting an interplay with adaptive immunity. IL-21 has the potential to directly activate NK cell proliferation and cytotoxic function, mediated by IL-21 receptor signaling expressed by these cells (39). In support of the possible interaction between innate and adaptive immunity mediated by NK cells, *in vitro* studies with human NK cells showed a selective expansion of an NK cell subset co-expressing CD86 and HLA-DR and lacking NKp44, as the result of IL-21 stimulation. In turn, co-stimulation of human naive CD4+ helper T cells by HLA-DR+ NK cells induce the differentiation of uncommitted central memory T cells (CXCR3+CCR6-CCR4-CXCR5-) (40).

Accordingly, a strong correlation of NKp30 expression with IL-21, IL2, IL-12, IL-15, and IL-18 was found selectively in the SS SG with a higher degree of inflammation and ELS organization within the glands. T follicular helper (Tfh) cells are the main source of IL-21 and are indispensable for functional GC formation, B-cell activation, and antibody production (41). Our group has shown that IL-21 is produced by Tfh cells within ELS in the inflamed SS SG and supports B-cell autoreactivity (33). Taken together, one could speculate that the production of IL-21 within ectopic GC in the ELS of pSS could further support Nkp30 upregulation at an advanced stage of the disease. In line with this hypothesis, we found that Rituximab, known to reduce ELS organization (42), was able to prevent NKp30 upregulation in the SGs of SS patients. Nonetheless, this observation comes from a relatively small number of patients and will require confirmation in larger cohorts.

pSS is the prototype of autoimmune disease associated with B-cell hyperactivity and autoantibody formation, which results from an aberrant response of adaptive immunity following tissue damage, self-antigen exposure, and recruitment of antigen-presenting cells. In the early stages of the disease, an initial insult, viral or environmental, is thought to cause EC apoptosis and mobilization of DCs, which pick up apoptotic cells, and activate T cells resulting in tissue damage (4).

The role of NK cells in this pathogenic process has not been clearly defined. Most studies focused on circulating rather than tissue-resident NK cells, mainly because of limited access to diseased tissues. Furthermore, studies on peripheral NK cells are confounded by the phenotypic and functional heterogeneity of NK cells, contributing to the contradicting results (15–17). This probably also reflects the potentially distinctive roles NK cells play at different stages of the disease as well as different patient cohorts. Moreover, the role of NKp30 cannot be explored in mouse models of pSS, as NKp30 is not conserved by murine NK cells where it exists only in the form of a pseudo gene (43).

By analyzing human salivary gland samples from patients with SS and sicca controls, we were able to localize NKp30+ cells in the SGs tissue at the periphery rather than within the inflammatory *foci*, where NKp46+ cell infiltration was also described (26) using NKp46 as a pan-NK marker. The comparison of the expression levels of *NCR1*/NKp46, *NCR2*/NKp44, and *NCR3*/NKp30 showed a higher expression of NKp30 in SS salivary glands; nevertheless, the localization of NK cells expressing different NCRs has never been compared in SS SG. Of note, the NKp30 ligand, B7/H6, was mainly expressed by SG EC, suggesting a direct interaction of NK cells with SG EC. B7/H6 has been hypothesized to be expressed by apoptotic SG EC (4), thus facilitating the direct interaction with NK cells, the activation of NK cells, and perpetuation of inflammation within the glands. However, no data have been reported on the mechanism leading to the induction of B7/H6. It showed to be induced in inflammatory conditions both *in vitro* and *in vivo* (44). *In vitro* studies on human SG cell line stimulated with inflammatory cytokines increased in SS SG, such as IL-17, IL-22, IL-23, or TNFα, showing that the upregulation of B7/H6 is mainly driven by TNFα (26).

Surprisingly, within SS SG, we found that B7/H6 also expressed in the cytoplasm of plasma cells. It is interesting to note that a previous study in a transgenic mouse model reported that B7 H (another member of the growing B7 family, ICOSL) on the plasma cell surface drives an increase in the number of plasma cells secreting antigen-specific, high-affinity, class-switched antibodies, as well as a corresponding increase in serum concentrations of antigen-specific antibodies (45). B7/H6 might promote similar roles in plasma cells in pSS SG; however, future experiments will be required to elucidate the exact function of B7/H6 expressed by plasma cells in SS SG.

NKp30 is also critical for the interaction of NK cells with DCs; engagement of NKp30 on NK cells with its ligand B7/H6 on DCs results in the production of IFN-γ and TNFα by NK cells and maturation of DCs and IFN-α production (46, 47). NK cells might play a role early in the pathogenesis of pSS *via* interaction with DCs, which results in DC maturation and initiation of the adaptive immune response. A mouse model of pSS also demonstrated an early influx of DC and NK cells in the SG following innate immune response (13).

Finally, this is the first study to investigate the effect of immunomodulatory treatments on NK cells and in particular on NKp30 expression in patients with SS. We showed that treatment with rituximab, which reduces the local inflammatory infiltrates within the SG, can also ameliorate the expression of NKp30 in the glands.

Overall, our observations in this study support a possible dual role of NK cells in the inflammatory process underlying the disease pathogenesis of SS: following an initial insult on the SGs, NK cells interact directly with apoptotic/damaged EC possibly through NKp30 and B7/H6, respectively, resulting in the production of pro-inflammatory cytokines/chemokines and influx of more effector cells. NK cells also interact with DCs and promote their priming and maturation, which in turn leads to further NK cell and T-cell activation and initiation of the adaptive immunity, with the recruitment of T and B cells and formation of local inflammatory infiltrates and ELS. This interaction could take place either within the inflamed salivary glands or in the draining lymph nodes. Further functional studies exploring the receptor/ligand interaction and the mechanisms involved in this process will be needed to better clarify the role for NKp30 expressing NK cells in the pathogenesis of pSS.

## Data Availability Statement

The RNA sequencing data presented in the study are publicly available, and can be found here: https://www.ebi.ac.uk/arrayexpress/, E-MTAB-10517. All the other data, different from transcriptomic data, are available from the corresponding authors on reasonable request.

## Ethics Statement

The study involving human participants and sample collection were reviewed and approved by the local Ethics Committee: LREC 05/Q0702/1 and LREC 17/WS/0172 - Rheumatology/Oral medicine clinic- QMUL. For TRACTISS Ethics approval and governance approval were obtained from the Leeds West Ethics Committee (ref. 10/H1307/99) and the Leeds Teaching Hospitals NHS Trust respectively. The patients/participants provided their written informed consent to participate in this study.

## Author Contributions

EP designed the study and performed the experiments. ES, SG, FR, DL, LF-J, RC, FCh, FCa, and ML contributed to the data analysis and presentation. MF, LQ, and AT provided help with and facilitated the collection and characterization of human samples. MB, SBo, DM, SBe, SV, and CP provided scientific insight and provided resources for this study. EP, SG, and MB wrote the manuscript. All authors contributed to the article and approved the submitted version.

## Funding

This work was supported by project grants from the Italian Foundation for Cancer Research (FIRC)- grant 14910 to EP, Medical Research Council (MRC)- grant N003063/1 to MB, Versus Arthritis UK - grant 21753 to EP, the Italian Association for Cancer Research (AIRC)- IG 91104 to DM and by the intramural research program of IRCCS Humanitas Research Hospital to DM.

## Conflict of Interest

The authors declare that the research was conducted in the absence of any commercial or financial relationships that could be construed as a potential conflict of interest.

## Publisher’s Note

All claims expressed in this article are solely those of the authors and do not necessarily represent those of their affiliated organizations, or those of the publisher, the editors and the reviewers. Any product that may be evaluated in this article, or claim that may be made by its manufacturer, is not guaranteed or endorsed by the publisher.

## Supplementary Material

Supplementary Figure 1 **(A)** Representative images of immunohistochemistry staining for B7/H6 in pSS labial salivary gland biopsy and **(B)** human tonsil. Arrowhead: B7/H6+ cells. **(C)** Representative images of double immunofluorescence for NKp30 (yellow) and B7/H6 (green) in human tonsil. Cell expressing both NKp30 receptor and its ligand localize mainly outside the germinal centre in human tonsil (positive control), close to each other. The dashed line delimit the germinal centre. LZ, Light Zone; DZ, Dark Zone.

Supplementary Figure 2 Assessment of the degree of infiltration of SG biopsies and ELS organization in pSS by IHC staining for T cells (CD3), B cells (CD20), the presence of follicular dendritic cell (FDC) network (CD21) and plasma cells (CD138). The histological identification of ELS in the labial SG biopsies is defined as at least one infiltrate with clear B/T cell segregation and presence of FDC within B cell aggregates, suggestive of germinal center presence.

Supplementary Figure 3 **(A)** Test for normal distribution, QQ plot, for NCR3 gene expression evaluated by qPCR. Cut-off of 0.05 for normality test. **(B)** Violin plots showing relative expression of NKp30 transcript from RNA extracted from total SG tissue from SS (n=13) and NSCS (n=20) patients. Mann-Whitney U t-test statistics. **(C)** NKp30 transcript expression in SS SG segregated by ELS presence in comparison to NSCS controls. Kruskal-Wallis test with Dunn’s multiple comparison correction. *p < 0.05, **p < 0.01, ***p < 0.001. NSCS, non-specific chronic sialadenitis; SS, Sjogren’s Syndrome; ELS, Ectopic Lymphoid Structures.

Supplementary Figure 4 **(A)** Spearman correlation analysis between *NCR1* (top row) and *NCR2* (bottom row) with *GZMB, PRF1, IFNG* and with **(B)** *IL12A, IL21, IL2, IL15 and IL18* gene expression levels from bulk-RNA sequencing of SG tissues. pSS patients (n=24) segregated for the presence of ELS. Spearman correlation coefficient, R and p value, *p < 0.05, **p < 0.01, ***p < 0.001, ****p < 0.0001. ELS, Ectopic Lymphoid Structures; neg, negative; pos, positive.
