# Supplementary figures and images for: NKp30 Receptor Upregulation in Salivary Glands of Sjögren’s Syndrome Characterizes Ectopic Lymphoid Structures and Is Restricted by Rituximab Treatment
Source: Front Immunol. 2021 Sep 14;12:706737. doi: 10.3389/fimmu.2021.706737 (PMC8477027; doi:10.3389/fimmu.2021.706737)

**Figure Supplementary 1**

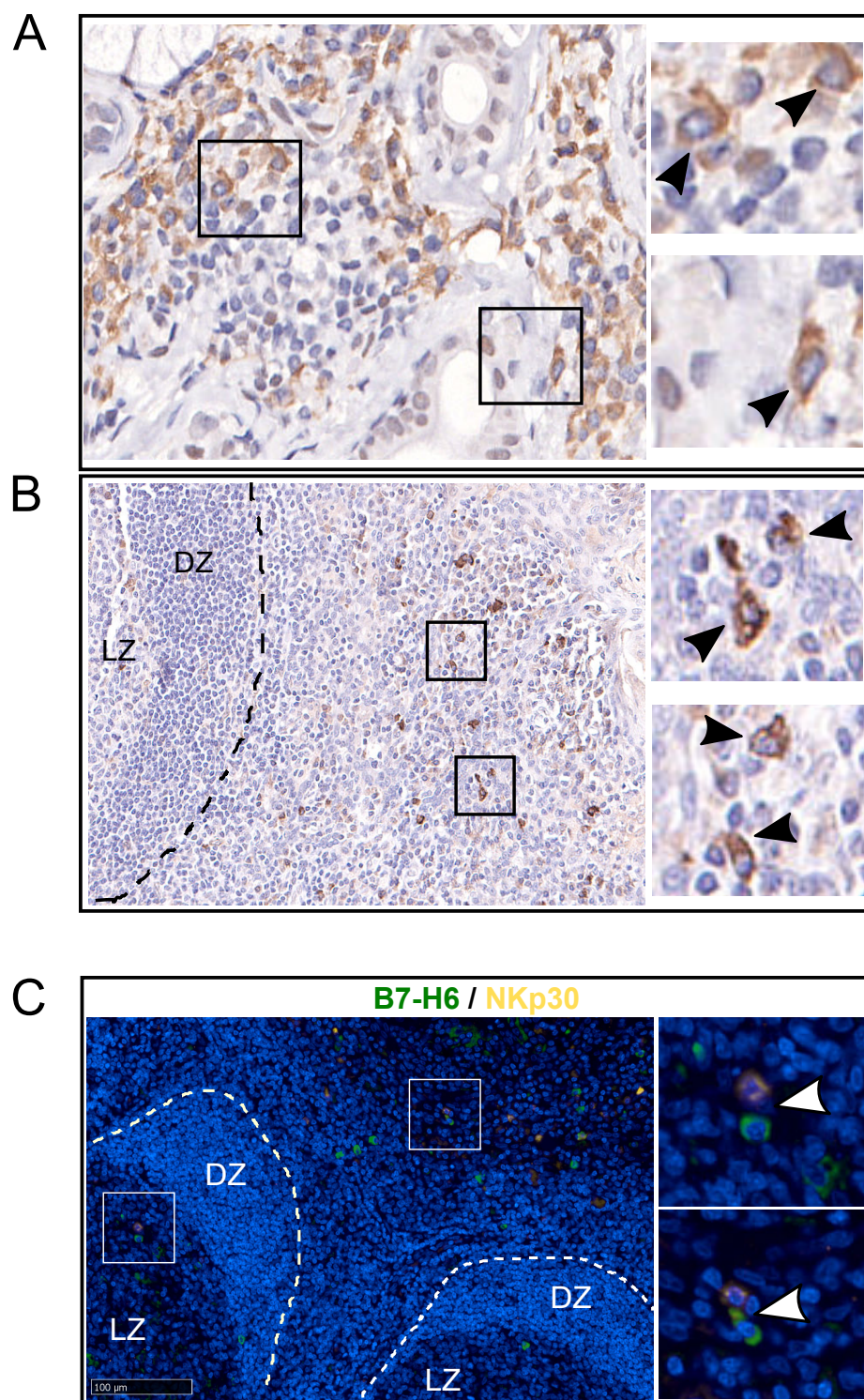

Supplement: Supplementary Figure 1 — (A) Representative images of immunohistochemistry staining for B7/H6 in pSS labial salivary gland biopsy and (B) human tonsil. Arrowhead: B7/H6+ cells. (C) Representative images of double immunofluorescence for NKp30 (yellow) and B7/H6 (green) in human tonsil. Cell expressing both NKp30 receptor and its ligand localize mainly outside the germinal centre in human tonsil (positive control), close to each other. The dashed line delimit the germinal centre. LZ, Light Zone; DZ, Dark Zone. [file Image_1.pdf]

**Figure Supplementary 2**

**A**

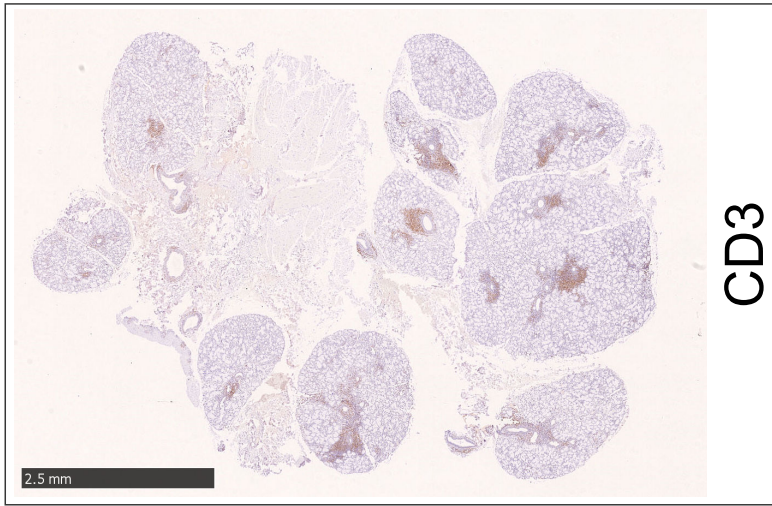

**B**

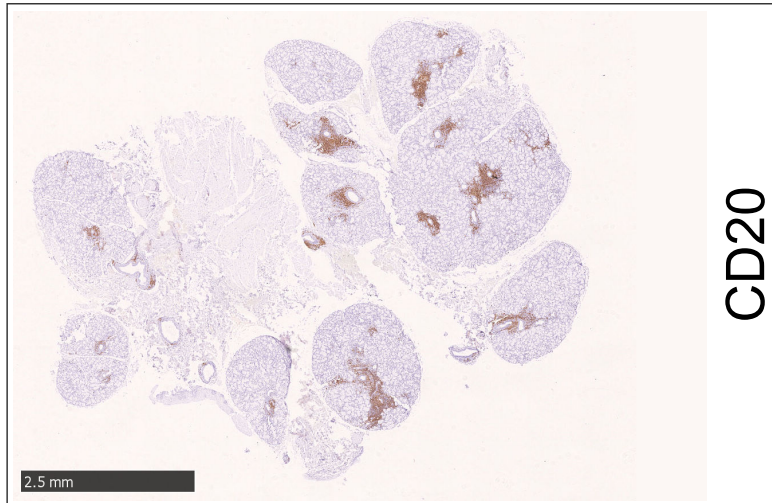

**C**

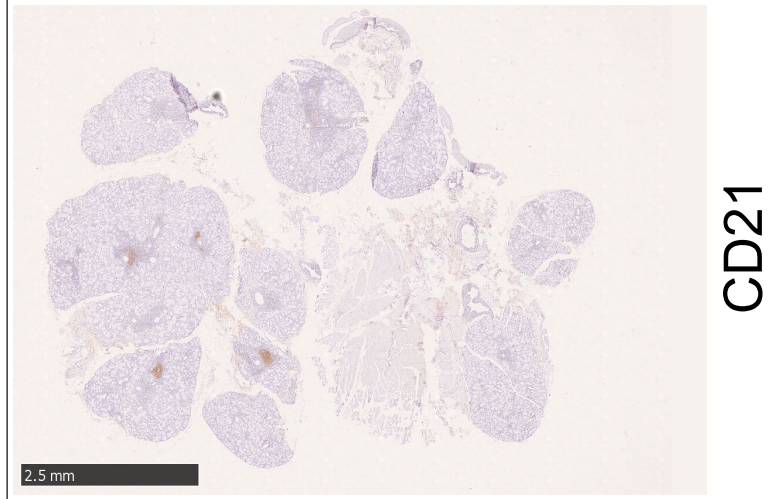

**D**

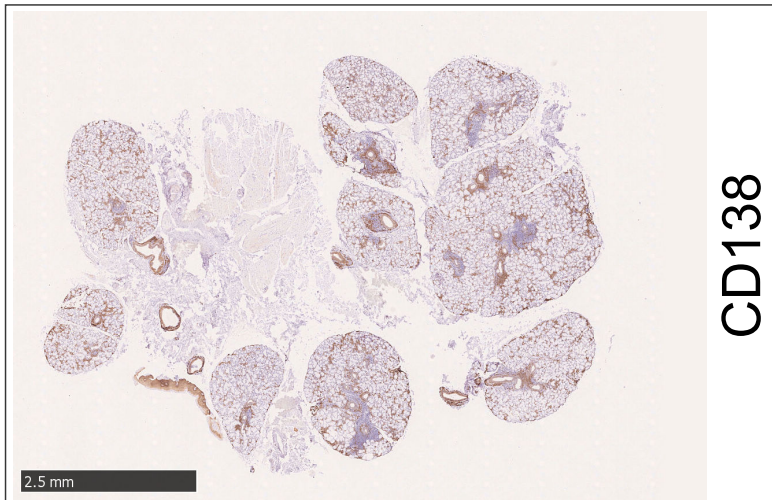

Supplement: Supplementary Figure 2 — Assessment of the degree of infiltration of SG biopsies and ELS organization in pSS by IHC staining for T cells (CD3), B cells (CD20), the presence of follicular dendritic cell (FDC) network (CD21) and plasma cells (CD138). The histological identification of ELS in the labial SG biopsies is defined as at least one infiltrate with clear B/T cell segregation and presence of FDC within B cell aggregates, suggestive of germinal center presence. [file Image_2.pdf]

**Figure Supplementary 3**

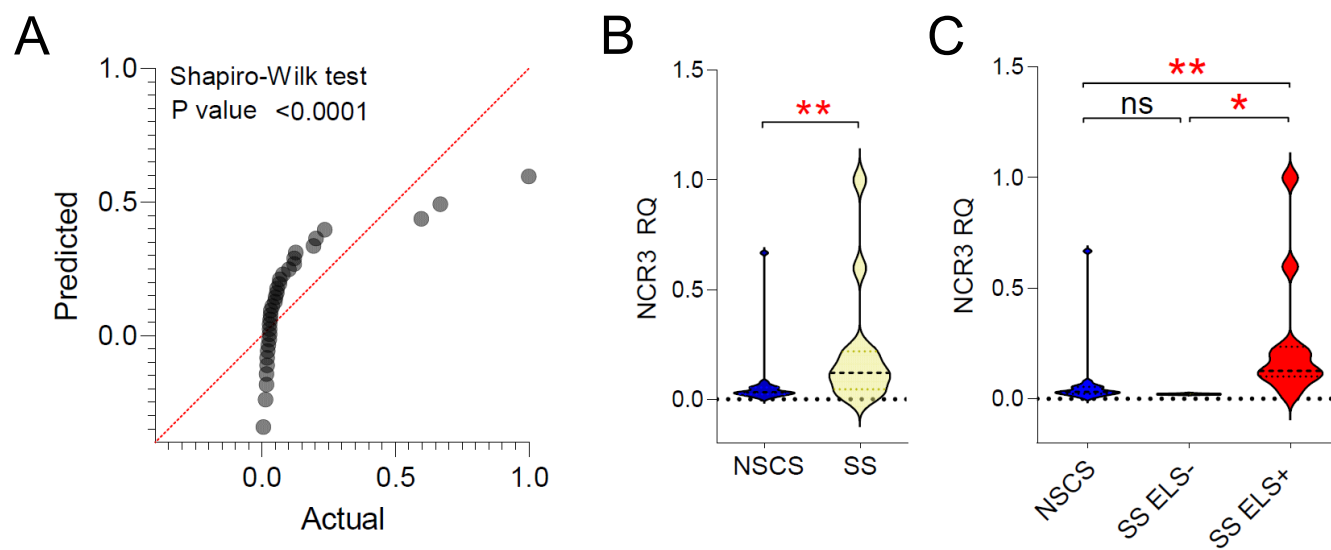

Supplement: Supplementary Figure 3 — (A) Test for normal distribution, QQ plot, for NCR3 gene expression evaluated by qPCR. Cut-off of 0.05 for normality test. (B) Violin plots showing relative expression of NKp30 transcript from RNA extracted from total SG tissue from SS (n=13) and NSCS (n=20) patients. Mann-Whitney U t-test statistics. (C) NKp30 transcript expression in SS SG segregated by ELS presence in comparison to NSCS controls. Kruskal-Wallis test with Dunn’s multiple comparison correction. *p < 0.05, **p < 0.01, ***p < 0.001. NSCS, non-specific chronic sialadenitis; SS, Sjogren’s Syndrome; ELS, Ectopic Lymphoid Structures. [file Image_3.pdf]

Figure Supplementary 4

A

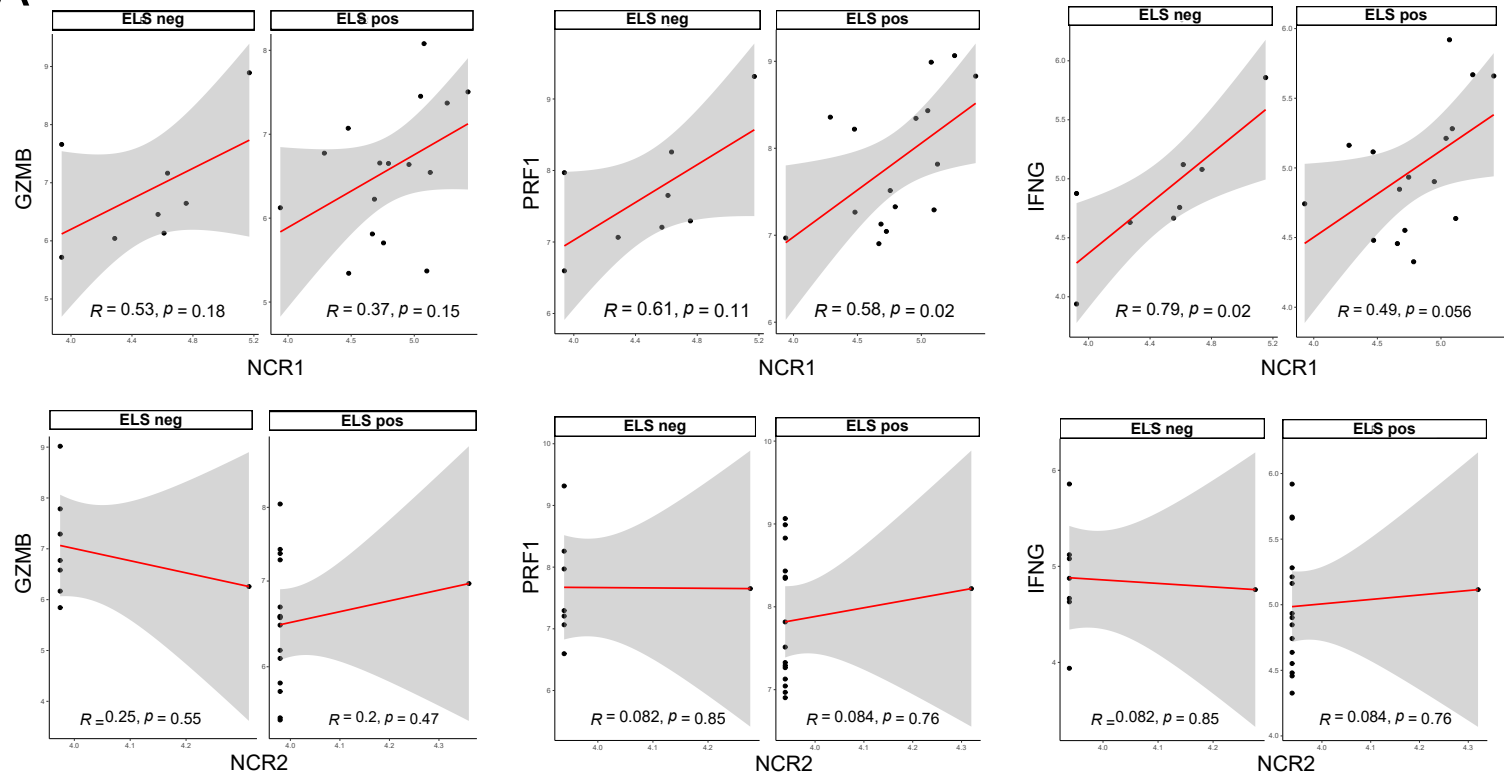

B

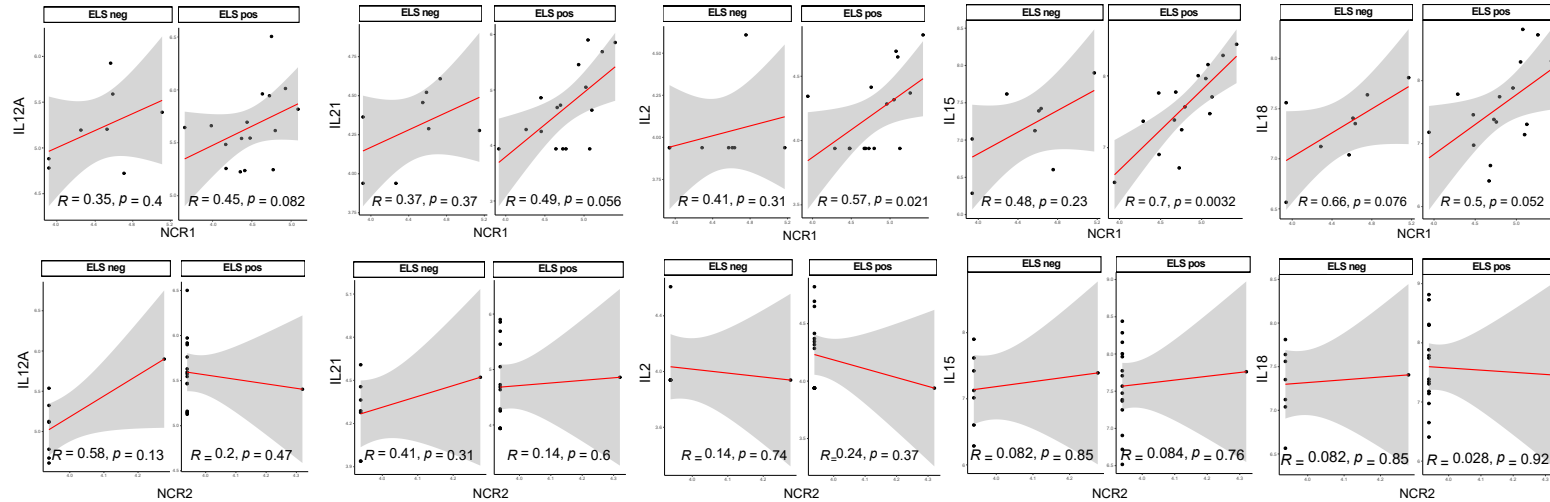

Supplement: Supplementary Figure 4 — (A) Spearman correlation analysis between NCR1 (top row) and NCR2 (bottom row) with GZMB, PRF1, IFNG and with (B) IL12A, IL21, IL2, IL15 and IL18 gene expression levels from bulk-RNA sequencing of SG tissues. pSS patients (n=24) segregated for the presence of ELS. Spearman correlation coefficient, R and p value, *p < 0.05, **p < 0.01, ***p < 0.001, ****p < 0.0001. ELS, Ectopic Lymphoid Structures; neg, negative; pos, positive. [file Image_4.pdf]
